# Articulatory, acoustic, and prosodic accommodation in a cooperative maze navigation task

**DOI:** 10.1371/journal.pone.0201444

**Published:** 2018-08-07

**Authors:** Yoonjeong Lee, Samantha Gordon Danner, Benjamin Parrell, Sungbok Lee, Louis Goldstein, Dani Byrd

**Affiliations:** 1 Department of Linguistics, University of Southern California, Los Angeles, California, United States of America; 2 Department of Linguistics, University of Delaware, Newark, Delaware, United States of America; Arizona State University, UNITED STATES

## Abstract

This study uses a maze navigation task in conjunction with a quasi-scripted, prosodically controlled speech task to examine acoustic and articulatory accommodation in pairs of interacting speakers. The experiment uses a dual electromagnetic articulography set-up to collect synchronized acoustic and articulatory kinematic data from two facing speakers simultaneously. We measure the members of a dyad individually before they interact, while they are interacting in a cooperative task, and again individually after they interact. The design is ideally suited to measure speech convergence, divergence, and persistence effects during and after speaker interaction. This study specifically examines how convergence and divergence effects during a dyadic interaction may be related to prosodically salient positions, such as preceding a phrase boundary. The findings of accommodation in fine-grained prosodic measures illuminate our understanding of how the realization of linguistic phrasal structure is coordinated across interacting speakers. Our findings on individual speaker variability and the time course of accommodation provide novel evidence for accommodation at the level of cognitively specified motor control of individual articulatory gestures. Taken together, these results have implications for understanding the cognitive control of interactional behavior in spoken language communication.

## Introduction

Convergence and entrainment are phenomena that have been observed in various types of interpersonal interaction, such as when two people walk in step [1], when musicians entrain to a common rhythm [2], or when the speech of conversing individuals becomes more similar to one another [3–6]. In each of these examples, people sometimes coordinate their behavior in service to some mutual goal, despite their lack of a single shared cognitive or neural system for achieving that goal [1]. Convergence (or accommodation) is the process, thought to be largely subconscious, by which individuals become more similar in their behavioral styles and/or properties. In the case of interacting speakers, studies have identified convergence effects in a variety of speech properties across linguistic levels. Acoustic convergence in speech has been observed for various phonetic properties, such as timing in acoustic onsets [7,8], speech rate [9–11], fundamental frequency [10], intensity [10], voice onset time [12], and vowel quality [13]. Still other studies have observed convergence in other properties of spoken language communication, such as choice of lexical item [14], choice of syntactic structure [15], direction of gaze [4], and body posture [16]. However, the basis of convergence in the *articulatory* domain and the articulatory and acoustic realization of convergence in *prosodic* patterning are not well understood. Recent studies have shown that articulatory properties of speech can also show convergence effects in measures of gestural stiffness [17] and articulator displacement [3], but these studies focused on word-level articulatory measures. Prosodically controlled connected speech materials have not yet been used in articulatory convergence research.

While convergence has been observed in many properties of spoken communication, there is still much to be understood about the mechanisms by which convergence is achieved, and why convergence emerges in interaction. At present, research shows that there are a variety of different ways in which individuals might change their language behavior when they are interacting as compared to situations of solo speech or speech immediately following an interaction [18]. Additionally, each speaker in an interaction may converge to different degrees, depending on a variety of factors—for example, on social status, attentional effects, task difficulty and/or cognitive workload, task setting, or on their role in the interaction [11,19–25]. Indeed, in some cases, divergence, rather than convergence, has been observed [26,27]. The research presented here uses a quasi-scripted task to evaluate the effects of prosodic convergence (or divergence) in both the articulatory and acoustic domains, including how these effects may vary across speakers.

Previous speech convergence studies have generally focused on quantitative measures of the *acoustic* properties of speech [6,8,10], but, as discussed by Tiede & Mooshammer [17], these acoustic measures are intrinsically related to the underlying motor behavior; indeed, they are the direct consequence of the goal-directed constrictions of the vocal tract. However, this relationship is not linear. Thus, an examination of speech articulator movement and/or articulatory patterning for speakers engaged in a dyadic speech activity will serve to illuminate convergence at the level of speech production control. If convergence is observed in some acoustic property, for example, duration, there must necessarily be some change in the motor behavior of one or both speakers that creates this convergence in the acoustic signal. However, more than one kind of motor adjustment may give rise to the acoustic convergence. If we find that the speakers converge in the motor control parameters driving the kinematics of articulatory gestures, this would indicate that speakers are able to accommodate at the level of cognitive/motor control. The present study will address the acoustic and motor aspects of convergence.

This study is possible because an instrumental set-up is now available that can track internal lingual movements simultaneously for two speakers face-to-face in a room [28]. We believe that this and Vatikiotis-Bateson et al. [3] (with one dyad) are the first such published studies of articulatory convergence for dyads in conversation, with works including [7,14,19 (the latter with 6 dyads)] paving the way. The present study uses movement tracking of speech articulators to examine whether convergence (or divergence) effects are present in local kinematic properties of speech gestures and their control parameters and whether such convergence between speakers persists for a brief time after a conversational interaction, as has been suggested in previous acoustic convergence research [18] (see also [29] for persistent post-interactional effect in finger movement coordination). Additionally, through careful control and manipulation of prosodic structure in the speech of the dyad via quasi-scripted stimuli, we consider the impact of higher-level phonological structure–such as prosodic structure–on the intonational and articulatory patterns that speakers exhibit in conversation and on the specific spans of the speech during which convergence is facilitated (cf. [10,30–35]). Specifically, we ask: How do prosodic properties of speech such as boundary strength, choice and excursion of boundary tone, and phrase-final lengthening differ when a speaker is engaged in an individual speech task versus when two speakers interact in a cooperative task? And, if speakers converge toward a prosodic pattern during the cooperative task, is the pattern maintained at all after the task has concluded?

Numerous studies have shown that prosodic structure, such as phrasal organization and salience (e.g., pitch accent), affects various articulatory and acoustic aspects of speech [e.g., 8, 19–23]. Phrase-final lengthening in both acoustics and articulation is a particularly familiar example. However, there is little information on how phrase- and utterance-level prosodic convergence between two speakers is reflected in their speech articulatory behavior, particularly because few convergence studies sufficiently control prosody in speech materials [36] (but cf. [5] *inter alia* and [37]), and even fewer have articulatory data on convergence [3,17]. The scarcity of prosodically controlled articulatory and acoustic data has made it difficult to investigate larger questions of prosodic convergence, such as how prosodic salience affects the emergence and/or amount of convergence in interacting speakers. In addition, some recent results [38] have been argued to show that final lengthening and the triggering of boundary tones (and also pauses) are all different consequences of a single prosodic- (pi-) gesture and its magnitude. For weak pi-gestures, only lengthening is triggered. As it increases in strength, a boundary tone and then a pause is triggered. If speakers converge at the level of abstract pi-gestures, then interacting dyads should show evidence of convergence, simultaneously in both boundary tones and final lengthening behaviors. The research described here therefore aims to provide novel data on convergence in prosodically controlled speech materials, with the goal of better understanding the characteristics of prosodic convergence—both boundary tones and final lengthening—in interacting speakers.

The impetus for interacting speakers to converge in speech properties is not fully understood. Some researchers have proposed convergence as a mechanism for aiding social interaction [13,18,29], while others see it as a partially automatic, low-level behavioral phenomenon [12,39]. These two possibilities are not mutually exclusive, and some researchers have noted that social and motoric bases for convergence necessarily interact [23,40]. Still other researchers propose that convergence cannot be low-level or automatic because speakers in conversation do not always converge equitably, nor do speakers merely imitate their conversational partners [21,41,42]. In assessing the impetus for convergence, it may be illuminating to look at the time course of when/if convergence behavior emerges during the course of a dyadic interaction. As yet, systematic study of the time course of convergence is not widespread [10], although some seminal works in this area do investigate either pre- or post-task speech of individuals [18,43]. In those works that focus on the time course of convergence, there is some evidence found for *persistence*, wherein features of speech that converged between speakers while interacting remain present in the speech or finger movements of one or both of the individuals after they are no longer interacting with one another [18,29,43]. The study presented here systematically investigates the time course of convergence with pairs of speakers from before they begin conversing, through the conversation, and after the speakers conclude their conversational task, with the speakers remaining together in a room continuously through these portions of the study.

## Materials and methods

### Setup and stimuli

#### Subjects and briefing

The University of Southern California University Park Institutional Review Board approved this research (UP-06-00138). Potential participants were recruited via flyers posted in public spaces around the USC campus, and those who responded were screened by study personnel for eligibility. Eligibility criteria included being at least 18 years old, being a native speaker of American English with normal speech and hearing, having no permanent metal dental work or metal in/around the mouth, and having no latex allergies (due to the latex gloves used for sensor placement). Participants were paired by study personnel into dyads of the same sex, and the dyad members were previously unfamiliar with one another and arrived separately to the site. Two study personnel and an equipment operator were present at each experiment. Before being briefed, participants were given a document containing information about the study and their rights as subjects and were asked to read the document and ask any questions, and could opt out of the study with no penalty if they chose. After each participant indicated their willingness to participate, one of the study personnel (always the same person) briefed the two participants together, ensuring that the participants met the aforementioned eligibility criteria and describing the equipment that would be used in the study. A total of four dyads (one male dyad referred to as Dyad S1-S2 & three female dyads referred to as Dyads S3-S4, S5-S6, & S7-S8, ages 18–32, mean age: 25) are included in this report (three additional dyads were not used due to recording failures or dialect misreporting).

#### Experiment setup

**Fig 1:**
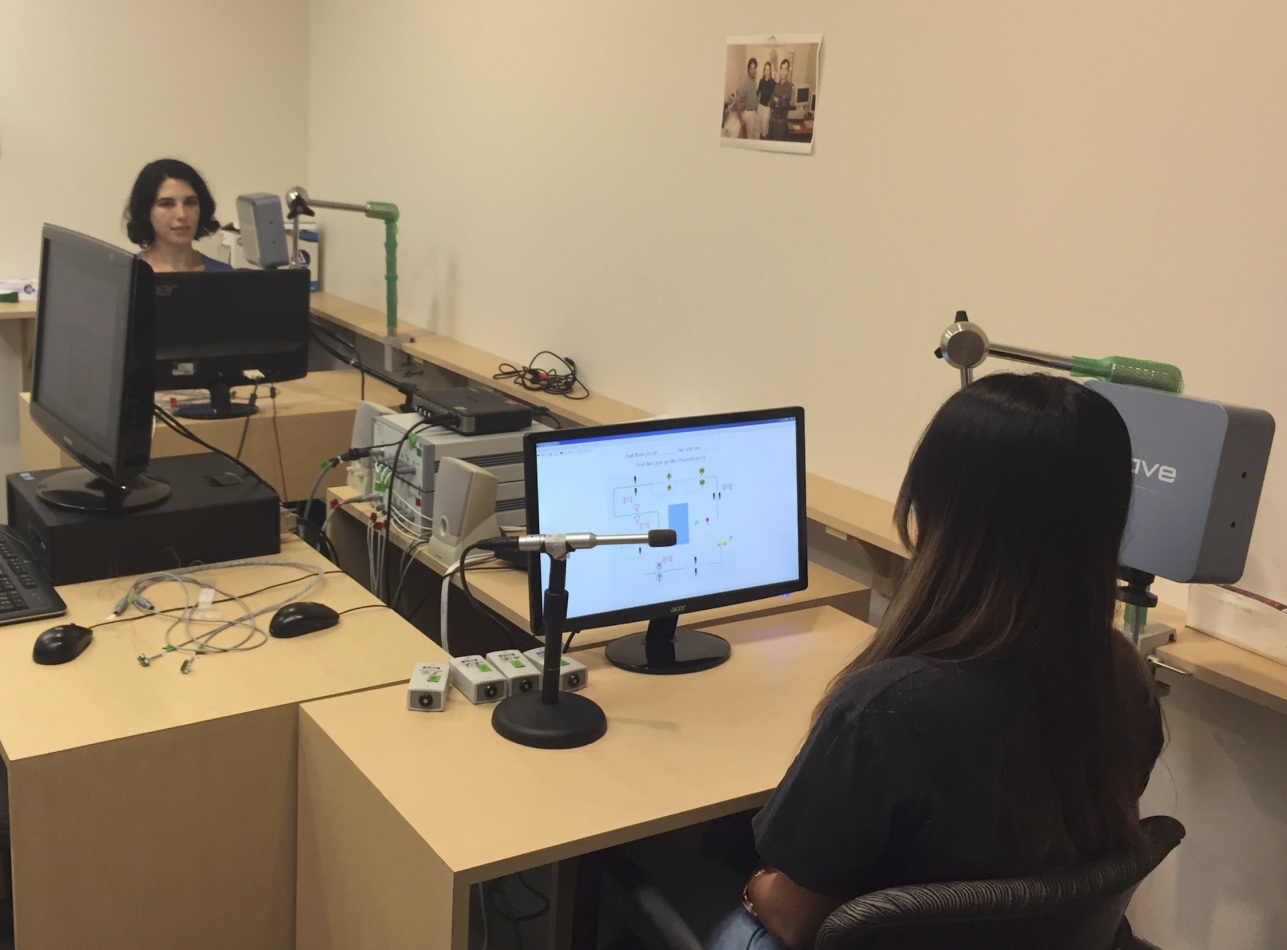
Articulography room setup. [The individual in this manuscript has given written informed consent (as outlined in PLOS consent form) to publish these case details].

This study was conducted in a sound-insulated room in the USC Phonetics Laboratory. Participants were seated, facing one another, at desks approximately two meters apart. Each talker had a computer monitor on their desk to view instructions and experimental stimuli (described below); the monitors were positioned so that the speakers had a clear line of sight to one another. The room organization is pictured in Fig 1. Each speaker was positioned beside a Wave electromagnetic articulography (EMA) system (Northern Digital, Inc.) with a high-quality tabletop microphone. The distance between the two subjects (i.e., the distance between the center positions of the two field generators) is about three meters. When a dyad member was participating in an individual response task, an opaque screen was placed between the two participants to block the line of sight between the dyad members. Additionally, the dyad member who was not participating in the individual response task listened to music during that interval using over-the-ear headphones to limit their ability to listen to the other dyad member’s speech.

**Fig 2:**
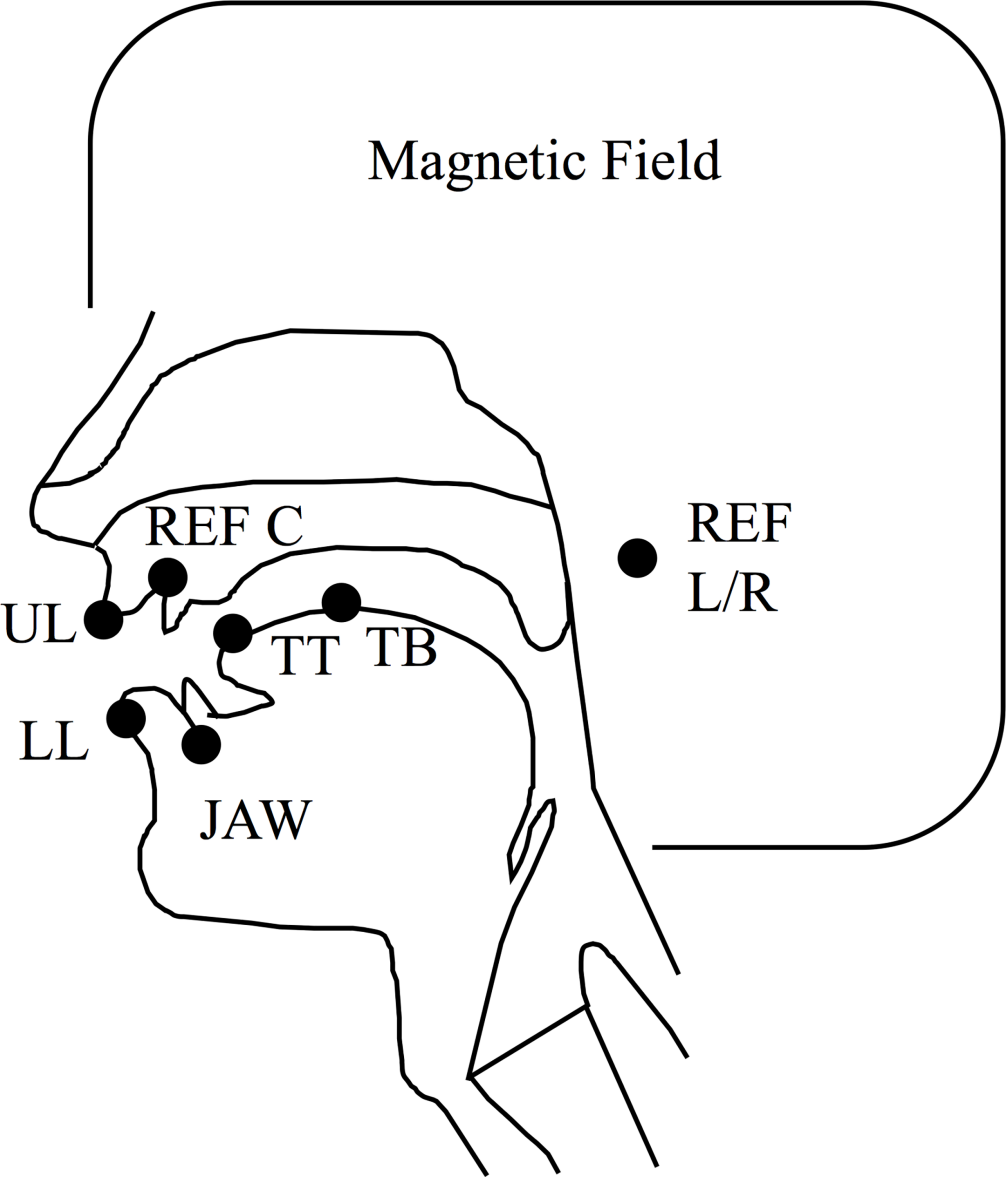
EMA sensor placement.

EMA uses sensor coils to track the movement of points on the head and the vocal tract. Each of the Wave systems has 8 sensor input channels available. After the briefing procedure, study personnel adhered sensor coils to each speaker’s face and vocal tract using a temporary adhesive; for expediency sensor placement was performed simultaneously on the speakers by two study personnel. Sensors were placed on the tongue tip, tongue body, jaw (lower incisor), lower lip, and upper lip. Additional sensors were placed on the upper incisor (center reference) and externally at the right and left mastoid processes (right and left references) to track head movement. Fig 2 shows approximations of the sensor placements on a mid-sagittal view of the vocal tract.

#### Stimuli

After participants were set up with the study equipment, both members of each dyad were briefed together on the format of the study by one of the study personnel. Participants were introduced to the types of tasks they would participate in, which included: reading sentences presented on their respective computer monitors, independently completing a maze task presented on their monitors, and completing a maze task cooperatively with one another. Before the study began, study personnel gave examples of how to complete each type of task, and at the beginning of each new task, speakers completed practice trials after which the study personnel gave feedback, if necessary, regarding how to complete that particular trial (for example, participants were corrected when they mistakenly used an incorrect target word in a maze task, or when they did not use the correct sentence frame for the target items, or when they started a maze in the wrong location). Individual tasks were repeated in two conditions. In the before condition, speakers had not yet interacted in conversation. In the during condition, speakers actively interacted with one another. In the after condition, speakers were no longer interacting together but had previously interacted with the other dyad member. The task presentation order is presented in Table 1.

**Table 1:** Stimuli presentation order.

Condition	Participant & Task
before	Speaker A individual sentence reading
before	Speaker B individual sentence reading
before	Speaker A individual maze task
before	Speaker B individual maze task
during	Speakers A & B cooperative maze task
after	Speaker A individual maze task
after	Speaker B individual maze task
after	Speaker A individual sentence reading
after	Speaker B individual sentence reading

The target words to be used in data analysis were matched across tasks and conditions. The words “beside” and “between” comprised utterance-medial targets; the words “lights” and “signs” comprised the utterance-final targets. In all cases, the medial and final targets were placed in a frame sentence to elicit a consistent prosodic phrasing pattern: “And then you go ___ [beside/between] the next two ___ [signs/lights].” The present report examines only “beside” and “signs,” as these targets share highly comparable articulatory trajectories word-finally ([bəsɑɪd]) and ([sɑɪnz]) in which tongue body raising occurring for the diphthong is followed by a tongue front constriction and release. Lastly, the utterance-medial “beside” was produced, as expected, without any pause or boundary tone. However, one speaker from Dyad S1-S2 often inserted a phrase boundary before or after “beside;” consequently, the nominal ‘phrase-medial’ condition was not analyzed for this particular dyad since the phrasal prosody varied from the expected pattern.

#### Sentence reading task

The sentence reading task was an individual response task used to allow comparison of each participant’s speech patterns when reading the target words in standardized sentence frame, to their behavior when the sentences are contextualized in a maze task. Subjects were asked to read eight randomized repetitions of each of the four sentences shown below.

1. *And then you go between the next two signs*.
2. *And then you go between the next two lights*.
3. *And then you go beside the next two signs*.
4. *And then you go beside the next two lights*.

As shown in Table 1, this individual sentence-reading task occurred both before and after the cooperative maze task. The reading task is not analyzed in this report.

#### Individual maze task

**Fig 3:**
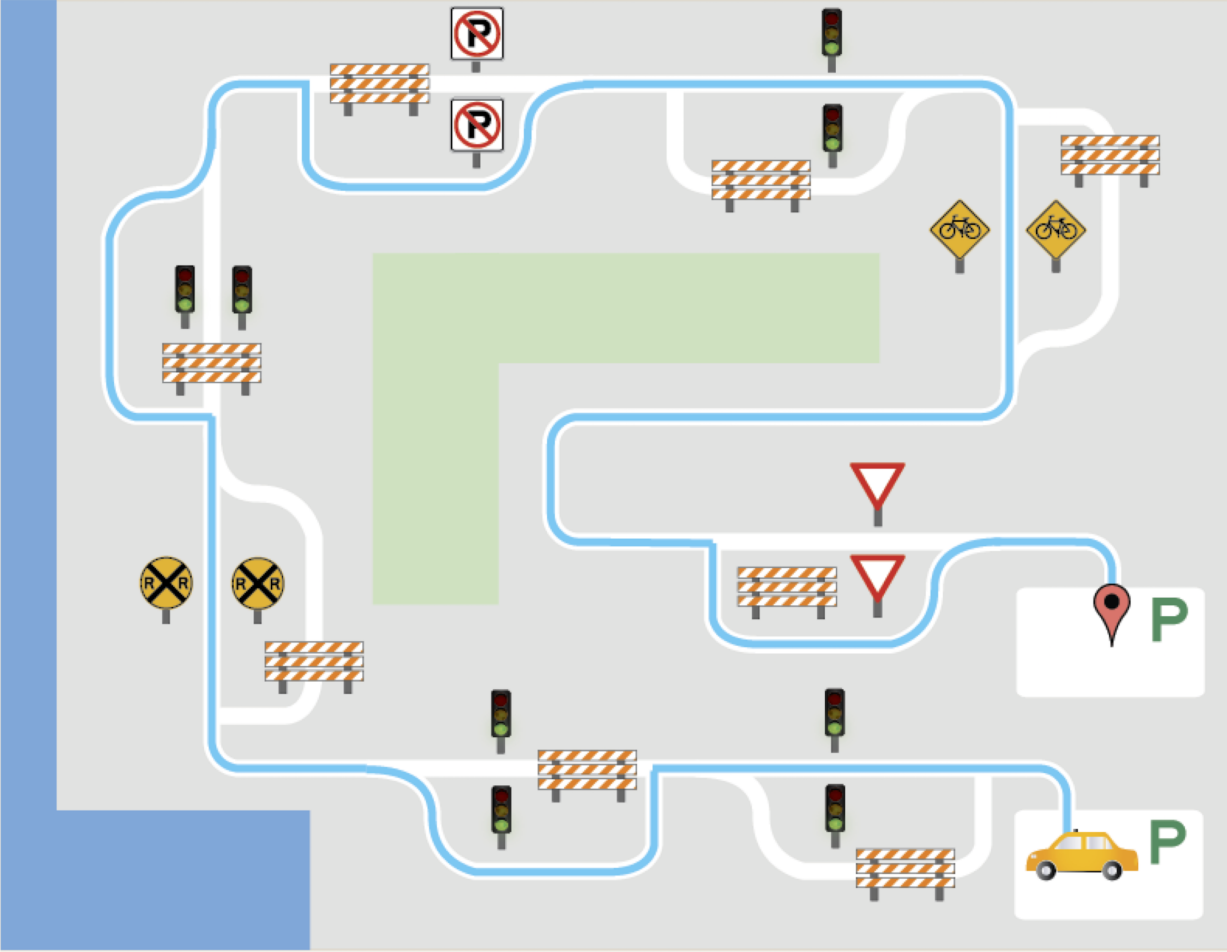
Example individual maze task.

In the individual maze task (see Table 1) the speaker sees an image similar to Fig 3, and, starting from the yellow car shown in a parking lot, they must follow the solid blue line through each of eight landmarks in the maze until the blue line reaches the red location marker in another parking lot. Each landmark can either be two signs or two lights. At each landmark, the speaker must use different target words within a frame sentence to describe that landmark. The blue line may run between two lights or between two signs at that landmark, or it may run beside (around to the side of) the two lights or signs at that landmark. The mazes were designed to have a balanced occurrence of the target word pairs (“between/beside” and “lights/signs”), and each maze was built on a common template with similar visual characteristics. Given this use of a graphic template and the requirement of like maze-landmark words, there is no reason to think that any significant variation in the difficulty of the mazes existed.

The speakers were instructed to start each new maze with the sentence “First you go on to the road.” At each landmark, the speaker must describe the navigation using the frame sentence described above: “And then you go ___ [between/beside] the next two ___[lights/signs].” The speaker must end the maze with the sentence “And then you go into the parking lot.” Each speaker completed two repetitions (one before and one after the cooperative maze task) of eight different versions of individual mazes presented in random order. If the speaker made an error in a maze, they were allowed to restart that maze.

#### Cooperative maze task

**Fig 4:**
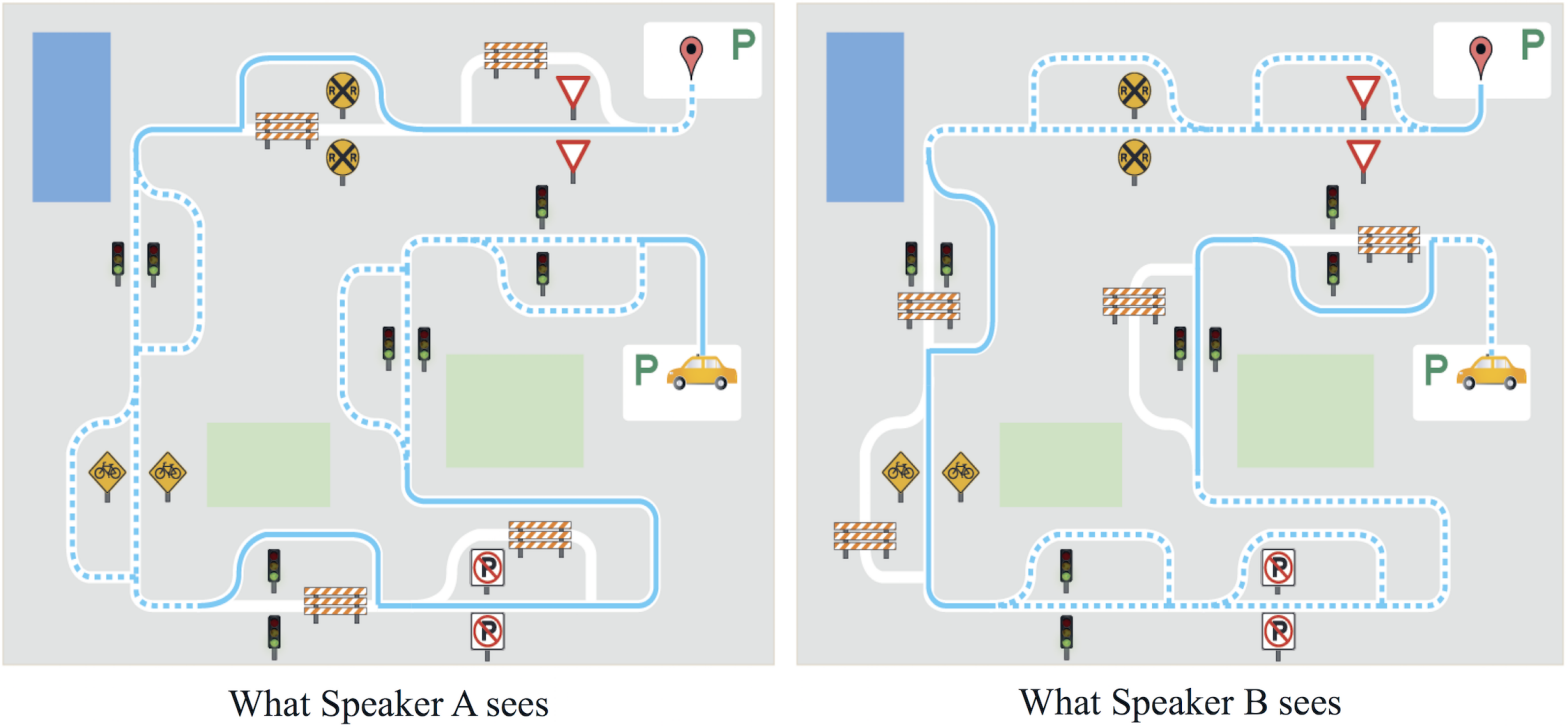
Example cooperative maze task.

The cooperative maze task required the participation of both dyad members. Each dyad member saw a version of the same maze on their respective monitors; however, some sections of the solid blue line were visible to one speaker, while other sections showed a dotted blue line passing both between and beside a landmark. The participants were instructed to navigate only those sections of the maze where they saw a solid blue line, as they had done with the individual maze, and to turn the floor over to the other dyad member to navigate the other sections indicated by dotted blue lines. An illustration of what each speaker sees in a cooperative maze task is shown in Fig 4. The solid blue lines in Speaker A’s view of the maze are replaced with dotted lines in Speaker B’s view of the maze, and vice versa. In the cooperative maze, speakers were required to navigate either one or two landmarks in a row before turning over the floor to the other speaker. Eighteen unique cooperative mazes were each presented two times at random to each dyad, for a total of 36 cooperative mazes. Speakers were allowed to start a maze over again if they made an error or spoke out of turn. In some cases, mazes had to be discarded due to recording errors, and in other cases additional mazes were inadvertently presented due to software control problems, resulting in between 34 and 41 cooperative mazes per dyad. A very few mazes in which an error occurred were discarded and restarted. In cases where a maze was restarted, speakers generally restarted within the first one or two utterances (of a maze with ten sentence production opportunities); one dyad had no restarted mazes, two dyads had one restarted maze, and one dyad had five. Only the final recording of each maze attempt was analyzed, and all mazes completed without error are included in the analyses.

### Data acquisition and post-processing

Two Northern Digital, Inc. (NDI) Wave electromagnetic articulography systems (Fig 1) for dual EMA data recording were controlled by NDI-provided data acquisition control software (Wavefront, ver. 2.0). The EMA trajectory sampling rate was set to 400Hz, and the speech sampling rate was fixed to 44.1 kHz. Audio data was recorded using high-quality Sennheiser condenser microphones (model E865S and model ME66) positioned on the desk near each speaker.

The presentation of speech material to the subjects used a custom-made Matlab script for stimulus presentation called Marta (Mark Tiede, Haskins Laboratories). The Matlab script reads an XML-formatted experiment design file provided by the user and presents speech material to the two subjects via start/stop recording buttons displayed to the experimenter in a graphical user interface (GUI). When the experimenter presses the recording start/stop button, the script interacts with a control application program interface (API) provided by the Wavefront software, and the current speech stimuli are simultaneously displayed to the two subjects. NDI provided a synchronization process for the two individual Wave units used for data collection based on a recorded impulsive, transient audio burst at the beginning of the dual recording session. The time lag (generally in the range of tens of milliseconds) in frames between the beginning of the impulsive sound in both recordings is used to align the individual recordings from each Wave unit.

The Wavefront software outputs sensor trajectories and acoustic speech data for each individual trial. To generate data appropriate for analysis, post-processing of the sensor trajectories is done using Matlab. First, any missing sensor position data are interpolated using the one-dimensional linear interpolation function (*interp1*) in Matlab, and then all trajectory data are low-pass filtered with a 20Hz cutoff frequency. Next, to correct for head movement, a reference coordinate system using the reference sensor position data was estimated, and then all sensor trajectory data were mapped to the reference basis coordinate system through a mathematical optimization scheme in order to minimize mapping errors due to sensor position measurement noise. Finally, the data were rotated to align the X-Y coordinates with the occlusal plane, measured at the initiation of data collection using an acrylic bite-plate with three sensors attached in a triangle shape.

### Measurements

#### Acoustically measured variables

Acoustic analysis was carried out using Praat software [44]. Praat’s built-in LPC tracking algorithm was used with fixed speaker-specific settings appropriate for each speaker’s voice, including creaky intervals, (i.e., pitch range [avg. ~60-avg. ~300] and voicing amplitude threshold [.2-.35]) that allowed for successful automatic pitch tracking for that speaker. Following this process, two authors with expertise in acoustic analysis both checked to confirm the accuracy of these measurements. All instances of the target words produced by each speaker were identified and labeled. The phrase-medial target word reported on here is “beside,” and the utterance-final target is “signs.”

**H% occurrence:** Pitch-tracking was used to calculate the pitch contour from which f0 maxima and minima in the targets were identified. If a speech error or unexpected pause occurred, that target was excluded. Boundary tones were measured in the phrase-final target word “signs.” Standards as designated in the Guidelines for ToBI Labelling [45] were used for labeling boundary tones. Two authors, experienced ToBI labelers, determined and labeled the boundary tones, which were then cross-checked by both authors. The sentences typically had a H% boundary tone, i.e., a continuation-rise intonation that indicates non-finality in a dialogue. Each speaker’s frequency of H% occurrence (expressed as a percent of all target utterances by that speaker) before, during, and after the dyad participated in the cooperative maze task was calculated.

**Frequency of boundary tone f0 peaks:** The utterance-final f0 maxima for the H% boundary tones in the final target word were measured for dyads S3-S4 and S5-S6. Dyads S1-S2 and S7-S8 used primarily non-H% boundary tones, exhibiting either a f0 valley or low plateau on the phrase-final syllable. For these two dyads, utterance-final f0 minima of non-H% boundary tones were measured.

**Sentence duration:** Acoustic sentence duration was measured in Praat for each utterance.

#### Articulatory variables

**Fig 5:**
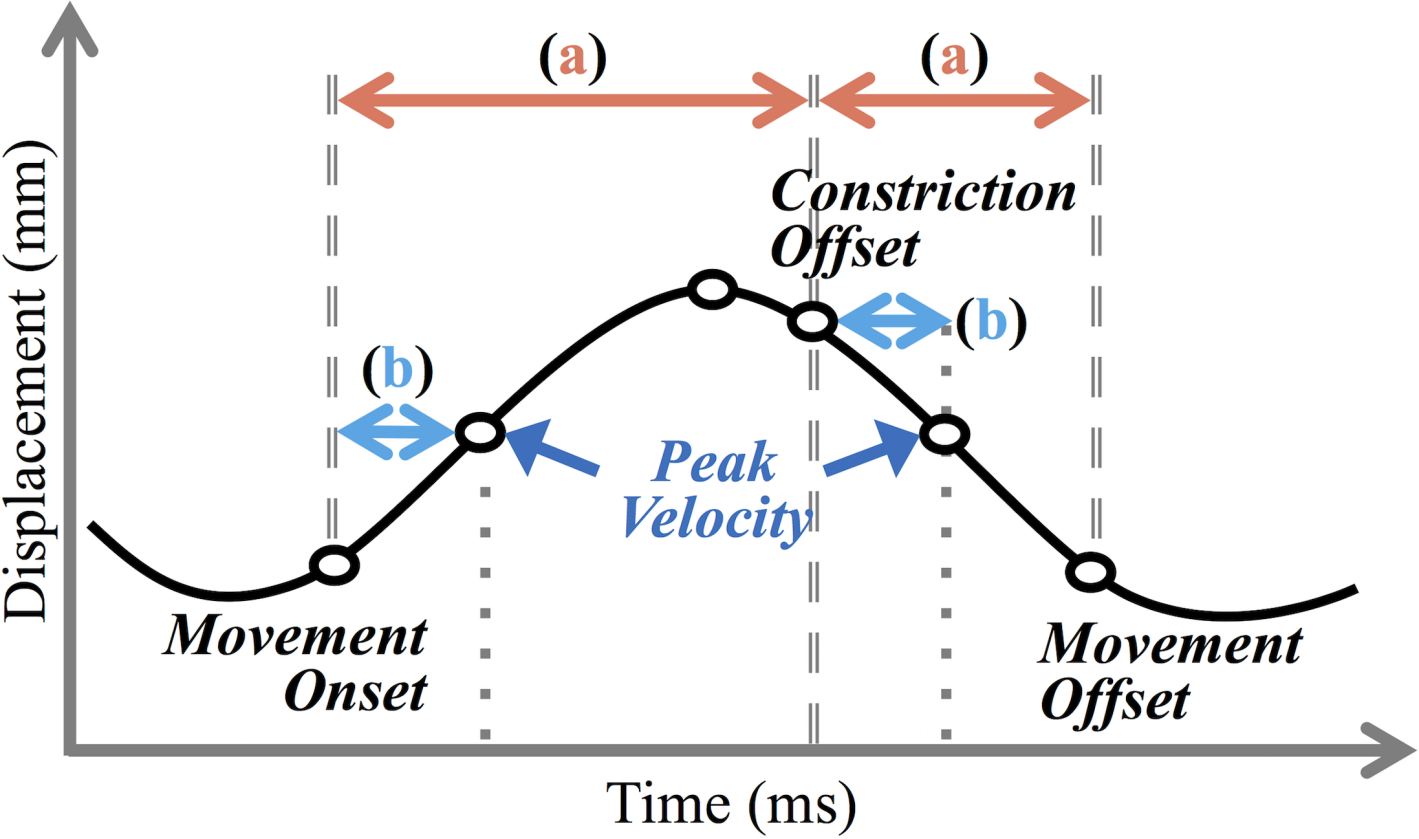
Example articulatory time function with measurement landmarks.

Articulatory analysis was carried out using MView (Mark Tiede, Haskins Laboratories) in Matlab. Kinematic time functions for tongue body and tongue tip sensors for the final tongue front constriction and release of the target word were analyzed. For all dyads except S3-S4, tongue tip *y*-trajectories were used. In Dyad S3-S4, tongue tip articulatory data was missing or unusable, so the tongue body sensor *y*-trajectory was used. (These two sensors were placed in close proximity on the tongue and we find little difference in their experimental behavior in this study.) The MView procedure *findgest* was used to label articulatory landmarks of interest (Fig 5). The procedure was parameterized to define movement onset as the point where the magnitude of the velocity first crosses a +/-10% threshold of the magnitude of the first velocity peak (the leftmost *b* in Fig 5) and movement offset as the point where the magnitude of the velocity falls below the same threshold of the second velocity peak. Constriction offset was identified as the first threshold-crossing point before the second velocity peak.

These temporal landmarks were then used to calculate several derived duration variables, described below.

**Movement duration [for constriction and release]:** The intervals labeled (a) in Fig 5—from the point of movement onset to articulatory constriction offset, and from constriction offset to movement offset—were defined as closure duration and release duration respectively. **Time-to-peak-velocity (TPV) [for constriction and release]:** The measures labeled (b) in Fig 5—the time from movement onset to closure peak velocity, and the time from constriction offset to release peak velocity—were defined as time-to-peak-velocity for the closure and for the release, respectively. TPV is an index of gestural stiffness, a temporal control variable for linguistic gestures [46].

### Data analysis

Because no uniform predictions could be made with respect to convergence or divergence, each dyad was quantitatively assessed independently. For each of the measures detailed above, this study investigates whether the dyad’s speakers exhibit *convergence*, *divergence*, and *persistence*:

- Convergence is indicated when measures become more alike in the during (cooperative) condition or in the after condition compared with the before condition. Convergence may be largely due to alterations made by just one of the two speakers, or may occur due to changes made by both speakers that cause the quantified speech measures for each individual to become more similar to one another.
- Conversely, divergence is indicated when measures become less alike in the during or in the after condition than in the before condition.
- Persistence (of either convergence or divergence) is indicated when convergence (or divergence) is found in both the during and the after condition.

In order to create appropriately paired data for analysis, each of the measures listed above, except for H% occurrence, was analyzed as follows: For each speaker (Speaker A or Speaker B), the values of the measure in the during and after conditions were averaged across the targets produced within each maze the speaker encountered (a total of 4 values per maze). As the maze presentation order is crucial for testing the between-speaker accommodation, this averaging scheme is essential for the during (and after) condition mazes. However, pairing values between speakers in terms of the presentation order in the before condition, when they have not yet interacted, is not necessary. Therefore, for the before condition, rather than averaging utterance measurements within each maze, we used the 32 individual values from each speaker of a dyad (4 utterances per maze x 8 before condition mazes). Thus, the basic data analyzed for a given measure for a given subject includes 32 values for the before condition, 34–41 values for the during condition, and 8 values for the after condition. For each measure, the maze values for the during and after conditions were arranged in the order the subject encountered them in the experiment. For articulatory variables, separate values were calculated for the target word in phrase-medial and -final positions. In addition, the absolute values of the differences (Δ, deltas) were calculated between the corresponding values of the members of a dyad. In the before and after conditions, for example, the value for the first maze encountered by speaker A was paired with the value for speaker B’s first maze, speaker A’s second maze value was paired with speaker B’s second maze value, etc. In this way, the between-speaker Δ values were calculated from corresponding time points in the experiment. With respect to the before condition, we created every possible pairing of values from the individual speakers regardless of the maze presentation order—each value from Speaker A’s 32 values is paired with each of Speaker B’s 32 values. This permutation results in 1024 paired values (32 x 32). The between-speaker Δ value from each pairing was then calculated, and a median value was obtained from the 1024 delta values, representing the between-speaker difference for the before condition. This median value derived from this large sample was then used to compare with the values obtained in the during and after conditions. If the Δ values for a given dyad decreased significantly between the before and during conditions or between the before and after conditions, convergence is said to have occurred. Conversely, an increase in the Δ values between the same conditions would indicate divergence.

Crucially, in addition to the above within-dyad speaker comparison, an additional ‘cross-dyad’ speaker comparison was performed to assure that a significant change of the Δ values between the before and during conditions or between the before and after conditions indeed stems from *accommodation* between interacting speakers, and is not solely driven by a general speaking style difference (i.e., solo speech vs. interactional speech). For this cross-dyad analysis, the values of each speaker (Speaker A or Speaker B) of Dyad *x* were lined up with the values of all speakers with whom they did not interact (i.e., the 6 speakers from the other 3 dyads). The same pairing scheme used to calculate within-dyad Δ values was then applied to calculate the Δ values between speakers from different dyads. For a given (within- or cross-) dyad, the condition-dependent shift in Δ values was calculated as follows: the difference between the median of the 1024 Δ values from the before condition and each of the Δ values from the during (or after) condition was calculated. The values of the *condition-dependent Δ shift* (i.e., |before Δ-during Δ| or |before Δ-after Δ|) were then averaged across the mazes in the during (or after) condition and used for within- versus cross-dyad speaker comparisons. If the within-dyad Δ shifts are larger than the cross-dyad Δ shifts, this serves as strong evidence of convergence (or divergence) between interacting speakers in a given dyad and evidence against such speaking adjustments being a purely stylistic effect due to the conversational interaction.

#### Significance testing

For the H% boundary tone occurrence, convergence was statistically evaluated by conducting a planned pairwise chi-square test of independence on the Δ value from each dyad, as the outcome variable was binary (H% vs. non-H%). The Δ value of H% occurrence was calculated as the difference between each speaker’s frequency of H% occurrence expressed as a percentage. The specific condition comparisons of interest involved before vs. during and before vs. after.

Condition-dependent shifts in Δ value (potentially indicating convergence or divergence) in the continuous acoustic and articulatory measures for a given dyad were statistically evaluated using the conservative non-parametric sign test, since the distributions of the measures were thought likely to depart from normality. For a given measure, the 34–41 Δ values from the during condition were compared to the median of the 1024 (32 x 32) Δ values of the before condition. If convergence were occurring, we would expect that more of the during values would be smaller (‘-’) than the median of the 1024 before values; whereas if divergence were occurring, we would expect that more of the during values would be larger (‘+’) than the median of the before values. The statistical significance of the asymmetry between ‘+’s and ‘-’s can be evaluated using a sign test. Similar tests of convergence and divergence were performed comparing each of the 8 after Δ values to the median of the 1024 before values.

In order to test whether significant condition-dependent Δ shifts could be due to accommodation between members of an interacting dyad, as opposed to a general shift in speaking style, the following analysis was undertaken. For a given dyad, whenever there was a significant condition-dependent Δ shift, its within-dyad *magnitude* was compared to the magnitude of shifts observed when those two speakers were paired with the 6 speakers with whom they did not interact (cross-dyad), using a one-sided sign test. The one-sided sign test is appropriate for testing the hypothesis that the shift in the Δ values from the non-interactional maze condition to the interactional maze condition (i.e., mean of |before Δ-during Δ| or |before Δ-after Δ| values) is consistently larger for within-dyad speakers than for cross-dyad speakers, suggesting accommodation. For most measures, pairing the values of Speaker A and Speaker B of Dyad *x* with the values from the 6 cross-dyad speakers resulted in a total of 12 mean Δ shift comparisons. In the case of the phrase-medial articulatory duration measures, from which one dyad was excluded due to an unexpected rendition of prosodic phrasing, a total of 8 mean Δ shift comparisons were made. For the utterance-final f0 peak measures, f0 maxima values from the two dyads who prevalently used H% boundary tones were paired up, and another pairing was done for f0 minima values from the other two dyads who used more non-H% boundary tones, each resulting in 4 mean Δ shift comparisons. Due to this small number of comparisons, the f0 peak measures could not be statistically evaluated using the sign test. Nevertheless, at least a marginal accommodation could be indicated, if *all* 4 Δ shift values are larger for within-dyad speakers than for cross-dyad speakers.

Finally, in order to test for the development of convergence or divergence over time in the during condition, a Spearman’s rho rank correlation method was conducted individually for each dyad and measure. The Δ values from the successive mazes of the during condition were rank-order correlated with the number 1 to ~38. A significant negative correlation indicates smaller deltas over time, and thus an increase in convergence, while a significant positive correlation would indicate an increase in divergence over time.

For all tests, the critical *p*-value was initially set to .05. In order to reduce the chances of obtaining false-positive results coming from multiple comparisons performed on a single set of data, the *p*-value was adjusted to a more stringent value using a Bonferroni correction. The corrections were made by dividing the critical *p*-value (.05) by the number of comparisons being made. In most cases, the adjusted *p*-values were .025, as there were two main comparisons (i.e., before-during and before-after). For the articulatory duration measures, *p*-values less than or equal to .0125 were considered significant, as there were 4 comparisons (2 maze-condition comparisons x 2 phrasal position comparisons).

## Results

The measurements can be divided into those that index the temporal structure of speakers’ utterances and those that index the intonational structure. Temporal measures include a global one: sentence duration (measured acoustically) and several local properties of the target word’s final constriction gesture (measured articulatorily): movement duration and time-to-peak-velocity (TPV) of both constriction and release gestures. Intonational measures include H% occurrence and f0 maxima/minima for boundary tone.

### Temporal measures

#### Sentence duration

**Fig 6:**
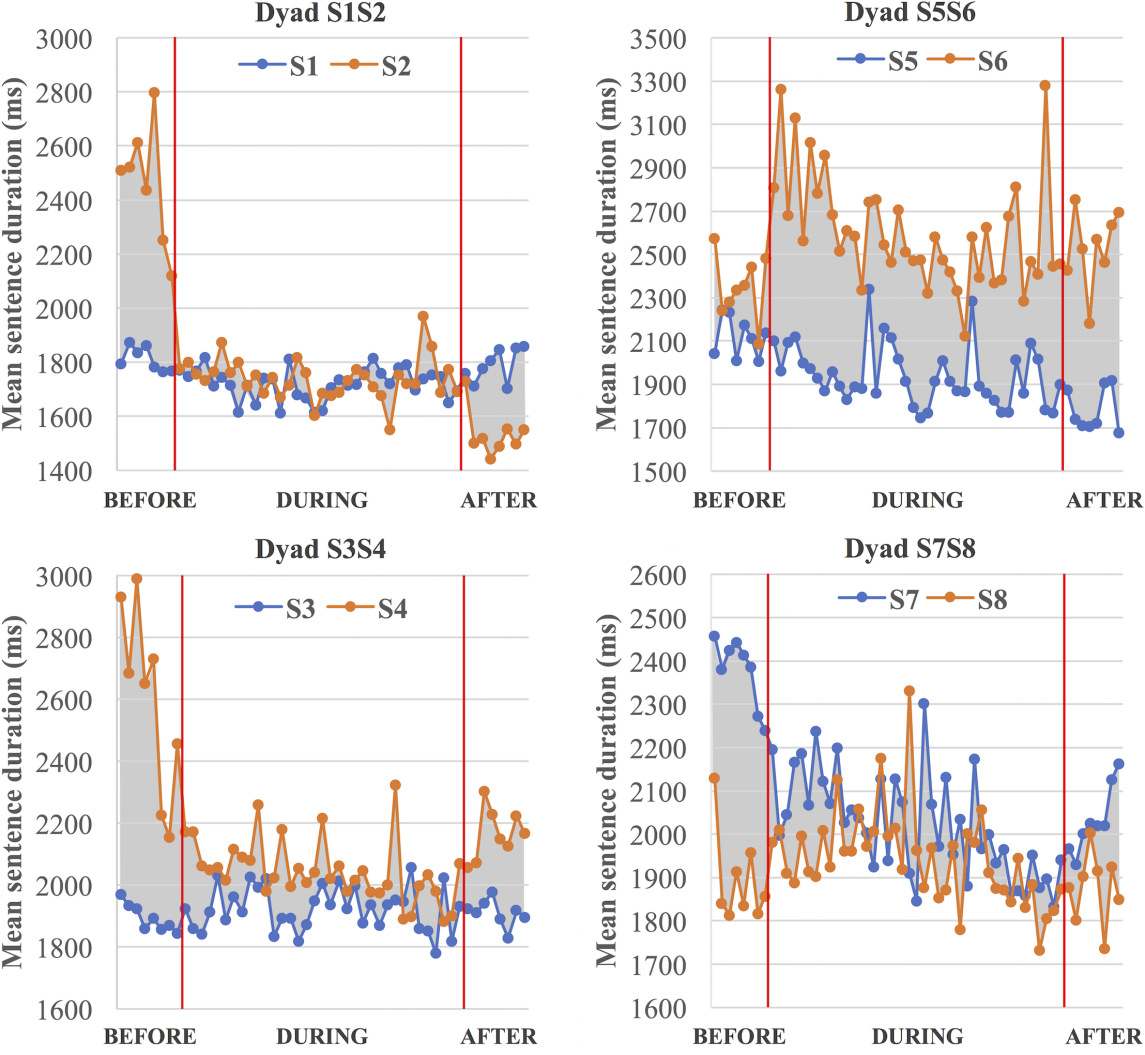
Mean sentence duration over the course of maze trials (before, during, and after the cooperative task) (all dyads).

To help visualize this and subsequent analyses, Fig 6 shows the results graphically for all four dyads. Each graph displays the sequence of measurements for each of the mazes, with the divisions between before, during, and after the cooperative task marked. One dyad member is shown in orange and the other in blue, and filling the area between the curves with gray serves to highlight the difference in value between the two speakers. Qualitatively, it is clear that three of the dyads (Dyads S1-S2, S3-S4, & S7-S8) show convergence during the cooperative task and after (when compared with the before condition), while Dyad S5-S6 shows divergence both during and after the cooperative task. Finding convergence or divergence in the after condition as well as the during condition indicates persistence. It is also apparent that convergence or divergence occurs nearly *immediately* in the during condition. Accommodation, when it occurs, does not appear to develop over time.

The quantitative statistical results support these qualitative observations and are shown in Table 2. The table shows median values for each condition for each speaker (columns 1 and 2) and the Δ measure (in column 3). For the all three converging dyads (Dyads S1-S2, S3-S4, & S7-S8), the sign tests comparing the values in the during condition to the median of the before condition and the values in the after condition to the median of the before condition are both significant (*before > during; *before > after). Likewise, Dyad S5-S6 shows statistically significant divergence for both comparisons (*before < during; *before < after). As indicated by a double asterisk (**) in Table 2, a one-sided sign test further shows that for both comparisons, the shifts in the Δ values are significantly larger when the speakers of a dyad are compared with one another (e.g., Δ values of S1—S2) than when those speakers are compared to speakers from different dyads (e.g., the 6 Δ values obtained comparing S1 to S3-S8 and the 6 Δ values obtained comparing S2 to S3-S8). This holds for all 4 dyads regardless of the direction of accommodation (**within-dyad Δ shifts > cross-dyad Δ shifts).

**Table 2:** Maze condition effects on sentence duration.

	**S1**	**S2**	**Δsentence duration**	***significance findings***
before	1781 ms	2382 ms	661 ms	
during	1728 ms	1738 ms	58 ms	** before > during
after	1792 ms	1511 ms	283 ms	**before > after
	**S3**	**S4**	**Δsentence duration**	***significance findings***
before	1887 ms	2570 ms	694 ms	
during	1924 ms	2033 ms	138 ms	** before > during
after	1915 ms	2159 ms	266 ms	** before > after
	**S5**	**S6**	**Δsentence duration**	***significance findings***
before	2120 ms	2323 ms	230 ms	
during	1907 ms	2556 ms	617 ms	** before < during
after	1732 ms	2548 ms	766 ms	** before < after
** **	**S7**	**S8**	**Δsentence duration**	***significance findings***
before	2373 ms	1844 ms	512 ms	
during	2004 ms	1945 ms	96 ms	** before > during
after	2020 ms	1890 ms	117 ms	** before > after

*sig.

**sig. and having *within-dyad Δ shifts > cross-dyad Δ shifts*.

Looking at the individual speaker contributions in the converging dyads, it is clear that most of the accommodation is accomplished by just one of the two speakers in a dyad. In some cases, only one speaker shows a change from the before condition in the direction of their partner; in others, both speakers do show some changes, but the magnitude of the change is *much* smaller for one of the talkers. For Dyad S1-S2, only Speaker S2 shows a change in the direction of convergence. Speaker S1 shows a small change in the diverging direction. For Dyad S3-S4, while both speakers show changes in the direction of convergence, the change for Speaker S4 is an order of magnitude larger than for Speaker S3. For Dyad S7-S8, both speakers again have changes in the converging direction, but the change for Speaker S7 is considerably larger than for Speaker S8. For the diverging dyad, Dyad S5-S6, both speakers have changes from the before to the during condition in the diverging direction, and the magnitudes of each speaker’s divergence are comparable.

#### Constriction duration

**Final position:** As shown in Table 3, Dyad S1-S2 again shows convergent Δ shifts in both the during and after conditions. Overall, the convergence is driven by both speakers, but Speaker S2 shows a larger change in the convergence direction in the after condition. In the after condition, the speakers’ values become significantly more similar to each other than to those of the rest of the speakers (**within-dyad Δ shifts > cross-dyad Δ shifts). Dyad S3-S4 shows convergence only in the during condition, driven by Speaker S4 in this case. Dyad S7-S8 also shows convergence in the Δ measure, but only in the after condition. The convergence is driven by Speaker S7 through during and after conditions; Speaker S8 shows a small change, but in the diverging direction. Dyad S5-S6 does not show any significant changes in the Δ measure.

**Table 3:** Maze condition effects on phrase-final constriction movement duration.

	**S1**	**S2**	**Δ****final** **constriction duration**	***significance findings***
before	133 ms	169 ms	43 ms	
during	138 ms	165 ms	24 ms	* before > during
after	143 ms	152 ms	10 ms	** before > after
	**S3**	**S4**	**Δ****final** **constriction duration**	***significance findings***
before	141 ms	159 ms	28 ms	
during	138 ms	142 ms	16 ms	* before > during
after	135 ms	154 ms	20 ms	
	**S5**	**S6**	**Δ****final** **constriction duration**	***significance findings***
before	155 ms	145 ms	60 ms	
during	166 ms	124 ms	50 ms	
after	191 ms	129 ms	49 ms	
** **	**S7**	**S8**	**Δ****final** **constriction duration**	***significance findings***
before	244 ms	150 ms	109 ms	
during	218 ms	140 ms	76 ms	
after	154 ms	121 ms	33 ms	* before > after

*sig.

**sig. and having *within-dyad Δ shifts > cross-dyad Δ shifts*.

**Medial position:** None of the dyads show significant convergence or divergence for constriction duration in medial position.

#### Constriction time-to-peak-velocity (TPV)

**Final position:** Table 4 shows constriction time-to-peak-velocity (TPV) results in phrase- final position. Dyad S7-S8 exhibits convergent shifts in the ΔTPV measure, for both during and after conditions, driven by Speaker S7. Dyad S1-S2 converges in the ΔTPV measure for the during condition, again driven by Speaker S2. Dyad S5-S6 shows a significant Δ shift in the direction of *convergence* but only in the after condition, driven by the change in Speaker S6’s behavior. It should be noted that there is no change in the during condition, and this dyad otherwise significantly diverges on the other temporal measures (i.e., sentence duration and phrase-medial constriction TPV). Lastly, Dyad S3-S4 shows no significant effect in ΔTPV in final position.

**Table 4:** Maze condition effects on phrase-final constriction TPV.

	**S1**	**S2**	**Δ****final** **constriction TPV**	***significance findings***
before	73 ms	66 ms	20 ms	
during	73 ms	69 ms	8 ms	* before > during
after	78 ms	64 ms	18 ms	
	**S3**	**S4**	**Δ****final** **constriction TPV**	***significance findings***
before	55 ms	76 ms	25 ms	
during	50 ms	63 ms	13 ms	
after	49 ms	70 ms	28 ms	
	**S5**	**S6**	**Δ****final** **constriction TPV**	***significance findings***
before	50 ms	33 ms	33 ms	
during	50 ms	33 ms	29 ms	
after	34 ms	38 ms	11 ms	* before > after
** **	**S7**	**S8**	**Δ****final** **constriction TPV**	***significance findings***
before	80 ms	66 ms	35 ms	
during	69 ms	61 ms	14 ms	** before > during
after	46 ms	47 ms	18 ms	* before > after

*sig.

**sig. and having *within-dyad Δ shifts > cross-dyad Δ shifts*.

**Medial position:** Table 5 shows that only Dyad S7-S8 converges significantly in the medial position TPV Δ measure in the during condition, driven by Speaker S7. Dyad S5-S6 shows significant divergence in the during condition, contributed by changes for both speakers. For both dyads (Dyads S7-S8 & S5-S6), the magnitudes of the accommodation effects are significantly larger for the within-dyad speakers than for cross-dyad speakers.

**Table 5:** Maze condition effects on phrase-medial constriction TPV.

	**S3**	**S4**	**Δ****medial** **constriction TPV**	***significance findings***
before	58 ms	65 ms	20 ms	
during	55 ms	60 ms	16 ms	
after	58 ms	65 ms	16 ms	
	**S5**	**S6**	**Δ****medial** **constriction TPV**	***significance findings***
before	38 ms	58 ms	25 ms	
during	21 ms	67 ms	44 ms	** before < during
after	29 ms	73 ms	43 ms	
** **	**S7**	**S8**	**Δ****medial** **constriction TPV**	***significance findings***
before	38 ms	30 ms	13 ms	
during	33 ms	32 ms	7 ms	* before > during
after	34 ms	31 ms	4 ms	**before > after

*sig.

**sig. and having *within-dyad Δ shifts > cross-dyad Δ shifts*.

#### Release duration and TPV

**Final position:** As shown in Table 6, Dyad S1-S2 converges significantly in the Δ constriction release movement duration measure in phrase-final position for the during condition. As in the other temporal measures, the convergence is driven by speaker S2. Dyad S7-S8 converges significantly in the Δ measure in the after condition, again driven by Speaker S7. Dyads S3-S4 and S5-S6 show no significant accommodation for this measure.

**Table 6:** Maze condition effects on phrase-final release movement duration.

	**S1**	**S2**	**Δ****final** **release duration**	***significance findings***
before	88 ms	106 ms	53 ms	
during	84 ms	53 ms	33 ms	* before > during
after	97 ms	125 ms	50 ms	
	**S3**	**S4**	**Δ****final** **release duration**	***significance findings***
before	81 ms	116 ms	45 ms	
during	100 ms	119 ms	31 ms	
after	116 ms	131 ms	27 ms	
	**S5**	**S6**	**Δ****final** **release duration**	***significance findings***
before	115 ms	90 ms	63 ms	
during	96 ms	84 ms	51 ms	
after	122 ms	79 ms	47 ms	
** **	**S7**	**S8**	**Δ****final** **release duration**	***significance findings***
before	178 ms	89 ms	90 ms	
during	176 ms	83 ms	92 ms	
after	128 ms	84 ms	47 ms	* before > after

*sig.

**sig. and having *within-dyad Δ shifts > cross-dyad Δ shifts*.

Table 7 shows release time-to-peak-velocity (TPV) results in phase-final position. Dyad S1-S2 again shows a significant convergent Δ shift in the during condition, driven by S2 as before. Speakers S1 and S2 converge more to each other than to the rest of the speakers (**within-dyad Δ shifts > cross-dyad Δ shifts). Dyad S3-S4 also converges significantly in the final TPV Δ measure in the during condition, driven by S4. No significant accommodation is found for Dyads S5-S6 or S7-S8 in this measure.

**Table 7:** Maze condition effects on phrase-final release TPV.

	**S1**	**S2**	**Δ****final** **release TPV**	***significance findings***
before	48 ms	61 ms	40 ms	
during	46 ms	28 ms	15 ms	** before > during
after	63 ms	72 ms	32 ms	
	**S3**	**S4**	**Δ****final** **release TPV**	***significance findings***
before	49 ms	60 ms	18 ms	
during	45 ms	54 ms	10 ms	* before > during
after	59 ms	64 ms	8 ms	
	**S5**	**S6**	**Δ****final** **release TPV**	***significance findings***
before	65 ms	70 ms	45 ms	
during	60 ms	55 ms	29 ms	
after	87 ms	51 ms	37 ms	
** **	**S7**	**S8**	**Δ****final** **release TPV**	***significance findings***
before	130 ms	49 ms	85 ms	
during	113 ms	59 ms	58 ms	
after	88 ms	60 ms	36 ms	

*sig.

**sig. and having *within-dyad Δ shifts > cross-dyad Δ shifts*.

**Medial position:** None of the constriction release measures in phrase-medial position show significant convergence or divergence for the dyads.

### Intonation measures

#### Phrase final intonation

**H% boundary tone occurrence:** As described in the Methods section, the analysis used a chi-square test on the Δ between the speakers in their overall percentages of H% used across the different conditions. Results are shown in Table 8. Dyads S3-S4 and S5-S6 converge in their choice of boundary tone when they were engaged in the cooperative task. The Δ values decrease significantly in the during condition when compared to the before condition (S3-S4: χ^2^(1) = 27.27, *p* < .025; S5-S6: χ^2^(1) = 17.5, *p* < .025). For Dyad S3-S4, the decreased between-speaker difference that occurs during the cooperative maze task continues in the after condition (before > after: χ^2^(1) = 31.25, *p* < .025). For both dyads, one speaker, not both, drives the convergence. Speaker S4 of Dyad S3-S4 and Speaker S6 of Dyad S5-S6 show increased H% occurrence in the during condition, becoming more like their partner in their boundary tone choice (S4: χ^2^(1) = 5.32, *p* < .025; S6: χ^2^(1) = 26, *p* < .025). Both dyads show that the speakers converge more to their interlocutors than to the speakers with whom they did not interact (**within-dyad Δ shifts > cross-dyad Δ shifts). Dyad S1-S2 shows significant divergence in ΔH% but only in the after condition (S1: before > after, χ^2^(1) = 33.79, *p* < .025; S2: before < after, χ^2^(1) = 5.6, *p* < .025). Oddly, both speakers show significant converging-direction changes in the after condition, but they overshoot in magnitude, resulting in divergence.

**Table 8:** Maze condition effects on H% occurrence.

	**S1**	**S2**	**ΔH% choice**	***significance findings***
before	59%	43%	16%	
during	48%	41%	7%	
after	10%	68%	57%	* before < after
	**S3**	**S4**	**ΔH% choice**	***significance findings***
before	100%	65%	35%	
during	95%	97%	3%	** before > during
after	95%	100%	5%	** before > after
	**S5**	**S6**	**ΔH% choice**	***significance findings***
before	94%	3%	91%	
during	77%	34%	43%	** before > during
after	100%	7%	93%	
** **	**S7**	**S8**	**ΔH% choice**	***significance findings***
before	21%	3%	18%	
during	20%	8%	12%	
after	10%	0%	10%	

*sig.

**sig. and having *within-dyad Δ shifts > cross-dyad Δ shifts*.

**f0 maxima/minima for boundary tone:** Dyads S3-S4 and S5-S6 used a preponderance of H% boundary tones, as shown in Table 8. For these two dyads, the value of the maximum f0 during that H% tone realization was taken as the relevant continuous intonational measure. For the other two dyads, the *minimum* f0 during the final L tone was taken as the relevant measure. Results were analyzed by sign tests as for the temporal measures and are presented in Table 9. All dyads except Dyad S1-S2 show some significant convergence effects in the Δ values: For Dyad S3-S4, convergence is found in the during condition, which persists into the after condition, fueled by Speaker S4’s accommodations. In all 4 dyad-dependent Δ shift comparisons, the convergent Δ shift is always larger for the within-dyad speaker pairings (Δ values of S3—S4) than for the cross-dyad speaker pairings (the 2 Δ values obtained comparing S3 to S5-S6 and the 2 Δ values obtained comparing S4 to S5-S6). Dyad S5-S6 shows convergence only in the during condition, caused by large changes in the direction of convergence by Speaker S6 (while Speaker S5 shows a smaller, divergent change). Dyad S7-S8 shows *divergence* in the phrase-final f0 minimum values in the during condition, driven by Speaker S8. Speakers S7 and S8 diverge more from their partners than from the speakers with whom they did not interact (for all 4 dyad-dependent Δ shift comparisons: within-dyad Δ shifts > cross-dyad Δ shifts).

**Table 9:** Maze condition effects on f0 peaks of boundary tone.

	**S1**	**S2**	**Δf0**_**min**_ **(non-H%)**	***significance findings***
before	46 Hz	82 Hz	36 Hz	
during	62 Hz	86 Hz	24 Hz	
after	72 Hz	79 Hz	13 Hz	
	**S3**	**S4**	**Δf0**_**min**_ **(non-H%)**	***significance findings***
before	226 Hz	118 Hz	114 Hz	
during	239 Hz	192 Hz	48 Hz	** before > during
after	233 Hz	164 Hz	67 Hz	**before > after
	**S5**	**S6**	**Δf0**_**min**_ **(non-H%)**	***significance findings***
before	252 Hz	164 Hz	88 Hz	
during	272 Hz	228 Hz	36 Hz	* before > during
after	275 Hz	106 Hz	163 Hz	
** **	**S7**	**S8**	**Δf0**_**min**_ **(non-H%)**	***significance findings***
before	189 Hz	167 Hz	23 Hz	
during	193 Hz	144 Hz	47 Hz	**before < during
after	193 Hz	131 Hz	51 Hz	

*sig.

**sig. and having *within-dyad Δ shifts > cross-dyad Δ shifts*.

[Note: Speaker S1 has an especially creaky voice and a tendency towards using L% boundary tones, resulting in an especially low f0 average.]

#### Development of convergence or divergence over time

**Fig 7:**
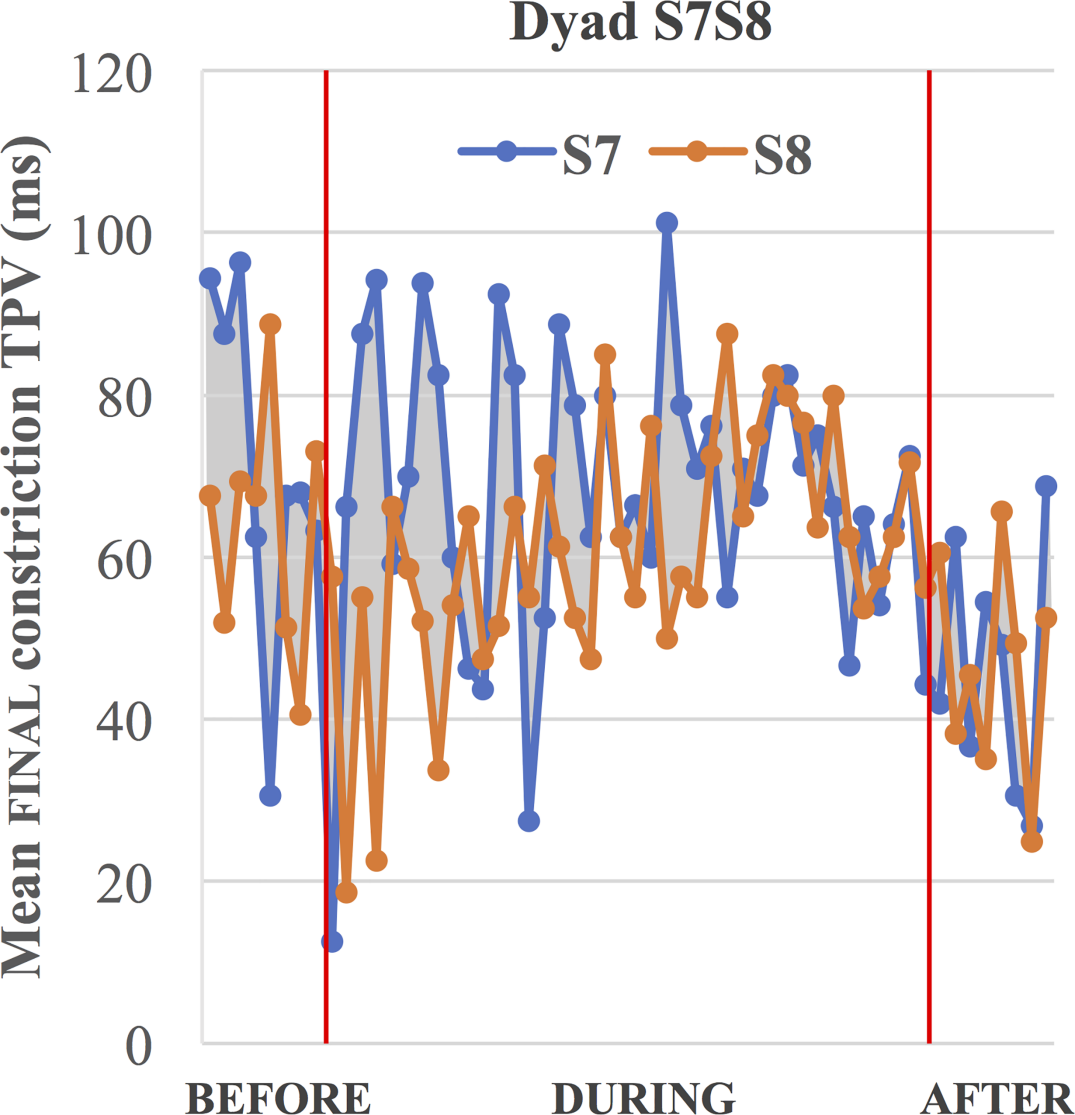
Dyad S7-S8’s mean phrase-final constriction TPV over the course of maze trials (before, during, and after the cooperative task).

Spearman rank-order correlation between trial (i.e., maze) number and the Δ values in the during condition was used as evidence of the buildup of convergence or divergence over the course of the experiment trials. A positive correlation would indicate more divergence on later trials, and a negative correlation would indicate more convergence on later trials. Only two measures showed significant correlations, both in the negative or converging direction. In sentence duration, Dyad S5-S6 shows a significant negative correlation, indicating that the Δ values decrease over the sequence of trials (*r*_*s*_ = -.33, *p* < .05). However, this is a dyad that we saw to diverge, not converge, from the before to the during condition. As can be seen in Fig 6, divergence occurs immediately in the during condition, but it is then followed by a tendency to lessen divergence later in the during condition. The other significant correlation was a negative correlation in constriction TPV (final position) for Dyad S7-S8 (*r*_*s*_ = -.57, *p* < .05). As shown in Fig 7, this dyad converges, so the results indicate a gradual increase in convergence over time. The same dyad shows a marginal negative correlation for sentence duration (*r*_*s*_ = -.28, *p* = .076), indicating increased convergence in the later trials, as can be confirmed visually in Fig 6.

## Discussion

This study of between-speaker accommodation presents a novel maze navigation task that allows for quasi-scripted but prosodically controlled speech from two interacting speakers. The goal of the study was to investigate inter-speaker accommodation—convergence, divergence, or persistence—in the articulatory and acoustic signatures of prosodic boundaries, with particular attention to evidence for (a) convergence at the level of cognitive/motor control parameters underlying individual speech gestures, (b) the possible independence of temporal and tonal signatures of prosodic boundaries, and (c) the development of accommodation over time, and its persistence after an interaction concludes. The primary finding is that convergence varies greatly among speakers and between dyads, as different speakers show patterns of convergence and divergence both while participating in the cooperative task *and* in a subsequent individual production task. However, within that broad landscape, there is evidence demonstrating *prosodic* accommodation in the motor control parameters of individual gestures, and also demonstrating the independence of temporal and tonal signatures of prosodic boundaries in convergence.

The results of the study are summarized in Table 10, which is a matrix of dyads *x* measures. Cells marked with ‘-’ showed evidence for convergence for the combination of dyad and measure, cells with ‘+’ showed evidence for divergence, and gray cells showed no accommodation effects. Numbers following the ‘-’ or ‘+’ indicate which of the subjects was primarily responsible for the accommodation in the Δ value (if both speaker numbers appear, then both contributed approximately equally). Parentheses ‘ ()’ are used to indicate significant accommodation results that were found *only* during a speaker interaction thus showing no persistence effects. The superscript “w>c” indicates significantly greater condition-dependent Δ shift for the within-dyad speaker pairings than for the cross-dyad pairings.

**Table 10:** Summary of results (dyads *x* measures).

	sentence duration	medial constriction duration	medial constriction TPV	medial release duration	medial release TPV	final constriction duration	final constriction TPV	final release duration	final release TPV	H%	f0_min/max_
**S1S2**	-2^w>c^	NA	NA	NA	NA	-2	(-2)	(-2)	(-2^w>c^)		
**S3S4**	-4^w>c^					(-4)			(-4)	-4^w>c^	-4^w>c^
**S5S6**	+56^w>c^		(+56^w>c^)							(-6^w>c^)	(-6)
**S7S8**	-7^w>c^		-7				-7^w>c^				(+8^w>c^)

- = convergence; + = divergence; gray = no effect; numbers indicate number of subject(s) driving accommodation; () = significant accommodation effects without persistence effects

^w>c^ indicates larger accommodation effects between within-dyad speakers than cross-dyad speakers.

Speakers in three out of four interacting dyads converge in sentence duration, which parallels previous reports on convergence in speech rate [9,10]. However, one dyad shows significant divergence, illustrating the kind of dyad-dependent variation that is possible in the tasks considered here [23]. One of the possible sources of accommodation in overall sentence duration could be the stiffness of the individual gesture control regimes, the control parameter that governs the amount of time required for a gesture to settle at its target value (which can be estimated as the time from the gesture onset to its peak velocity) [46]. This is also a cognitive parameter in that its value can be modulated by phonological structures, such as the distinction between vowels and consonants [47] or prosody. Dyad S7-S8 shows significant convergence of this parameter for constriction gestures in both medial and final position, thus providing novel evidence for accommodation at the level of cognitively specified motor control of individual gestures. Dyad S5-S6 shows a significant divergence of this control parameter, consistent with its divergence pattern in sentence duration. This is then another example of cross-speaker accommodation in a control parameter. The lack of an accompanying accommodation in the measured duration of the gestures that accommodate in stiffness is a bit puzzling, but it may result from the relative noisiness in measuring movement onsets and constriction offsets (which require a velocity threshold) compared with the robustness of measuring the time of a velocity peak, and/or it may relate to undershoot in the gesture reaching its underlying target value.

For the other two dyads, we do find evidence for accommodation effects in the articulatory durations that realize prosodic boundaries, which is an effect that has never been previously reported. Two dyads (Dyads S1-S2 & S3-S4) converged in the duration of phrase-final lingual closures—i.e., phrase-final tongue tip constriction movement duration. Dyad S1-S2 also converged in the stiffness control parameter (indexed by TPV) underlying the final gesture duration. Dyad S3-S4 was the only dyad that did not show any significant effect on the stiffness control parameter of a constriction gesture. In addition, these two dyads (Dyads S1-S2 & S3-S4) show some convergence in the temporal measures of the phrase-final constriction release. Dyad S1-S2 converges in final release duration and in its underlying control parameter. Dyad S3-S4 shows convergence in release TPV but not in release duration. No such effects are observed in Dyads S5-S6 and S7-S8.

Turning to the intonation measures, while previous studies have shown convergence in overall or phrase-final f0 patterning in dialogues (e.g., [10,43]), we provide a novel index of prosodic convergence, focusing on both categorical and continuous characteristics of phrase-final intonation. Interacting dyads that primarily use a H% boundary tone (Dyads S3-S4 & S5-S6) converge in their choice of the boundary tone, signaling with a H% boundary tone non-finality in a dialogue. Moreover, the speakers of these dyads also show accommodation in f0 peak values for these high boundary tones. The other two dyads, who produced principally low boundary tones, did not accommodate in their choice of boundary tone. The speakers of Dyad S7-S8 did, however, diverge in the value of the f0 minimum.

The experiment also affords an opportunity to test whether boundary tone and phrase-final lengthening, which are phonetic signatures of intonational phrase boundaries in English, are (potentially) cognitively executed independently of one another or whether both are governed by a single abstract aspect of phrasal structure. The accommodation effects reported here show variability across dyads but no support for the notion that boundary lengthening and boundary tones are linked to one another (cf. [38]). Two dyads (Dyads S1-S2 & S3-S4) show convergence in both lengthening and tones, but the other dyads show convergence in one of these measures but not in the other: Dyad S5-S6 shows convergence in intonation but divergence in lengthening; Dyad S7-S8 converges in final lengthening but diverges in tone. This finding is in line with a previous report on accommodation behaving differently in different measures [6,13,21], and it crucially further allows for the possibility that the intonational and temporal demarcations of prosodic boundaries can be independently realized in English. Why this independence obtains is worth further consideration. One speculative analysis of the behavior of the dyads that show apparent divergence in one domain of prosody (tone or rhythm) but convergence in the other is that the speakers are cooperating systematically, but on the divergent dimension they participate in—i.e., ‘cooperate’ by—forming an alternating (anti-phase) pattern (“you go high and I’ll go low.”).

While we have described significant condition-dependent Δ shifts as accommodation (either convergent or divergent), it is possible that such effects could result from individual speakers adopting a different speaking style during the interactive task (“conversational”) compared to that in the solo speaking task, and that there is no mutual accommodation *per se*. In our study, possible evidence in favor of this alternative is that in almost every case (except one, constriction TPV in final position for Dyad S7-S8), there is no statistically significant evidence of a linear trend of increasing accommodation over the duration of the interaction. Such a result would be expected if the speakers were not accommodating at all but rather changing speaking style modes from the very beginning of the during condition. Two lines of evidence argue against this interpretation, however. First, of the 18 significant condition-dependent Δ shifts in the during condition in Table 10 and 11 of them showed a significantly greater magnitude Δ shift than that exhibited by those two speakers when their data was paired with the data of the speakers with whom they did not interact (cross-dyad). This suggests that in at least these 11 cases, there is some dyad-specific accommodation going on, over and above any speaking-style differences. However, it does not completely rule out the possibility of the existence of a stylistic effect—the conversational pressures common to both speakers within a dyad—that leads both speakers to behave similarly [11,13]. Although our method was able to compare a speaker’s speech in a given condition to both within-dyad and cross-dyad talkers [10], the comparison between the speaker’s speech and the speech of their interlocutor in other speaking settings was not applicable in our study (cf. Cohen Priva et al’s [11] convergence study on speech rate exploiting such a method). Second, the results show many cases in which condition-dependent Δ shifts in the during condition persist into the after condition. Of the 18 significant effects in Table 10 and 9 cases show persistence. These findings accord with a previous report by Pardo [18], who found that acoustic phonetic changes made during a conversational task persisted to a recording session conducted immediately after the task. The after condition involves solo speaking once again, so the strong form of the speaking style analysis would not predict persistence effects in this condition. Moreover, there were 4 cases in which accommodation was found in the after condition but not in the during condition. Exactly how to account for this late or delayed accommodation is unclear, but, again, it is difficult to view the condition-dependent shifts in these cases as coming purely from speaking style adjustments with no such adjustments made during the interactional speech. To account for all the evidence, it is possible to hypothesize that there are indeed condition-dependent style changes, on top of which there are accommodation effects. The style shift changes could presumably occur early in the interaction, which could account for the lack of a significant time trend. But the accommodation serves to increase the magnitude of the condition-dependent shifts beyond that due to style shifts. Such increases may not be linearly related to elapsed time of interaction, which is why the correlations of maze number with Δ values were not significant. Future analyses of the time series data using alternative techniques may be revealing.

The results of the study also provide evidence that prosodically salient positions, such as preceding a phrase boundary, may be more sensitive to accommodation forces when speakers are interacting in a cooperative task. As shown in Table 10, of the 9 significant accommodation cases in the articulatory duration measures, 7 cases were phrase-final duration measures. Different dyads patterned differently, however: Dyad S1S2 showed significant convergence in all 4 cases of the phrase-final articulatory duration measures (but note that phrase-medial comparisons were not available for this dyad); Dyad S3S4 showed 2 significant convergence cases only in the final articulatory durations; Dyad S5S6 showed one case of a significant divergent shift in medial position; Dyad S7S8 showed significant convergence on constriction TPV in both medial and final positions. The results show that accommodation effects on gestural parameters are substantially more robust at phrase-edges (a prosodically salient position) than they are internally to phrases.

Variability in patterns of accommodation was one of the interesting findings of this experiment. Generally, one speaker of the dyad is particularly malleable; In Dyads S1-S2 and S3-S4, it is always one speaker who is responsible for the accommodation (S1 and S4, respectively). In Dyad S5-S6, Speaker S6 is always a major contributor to the accommodation, even though the accommodation is diverging in temporal measures but converging in intonation. Speaker S5 also contributes to some of the accommodation in temporal measures but not in intonation. Temporal convergence shown by Dyad S7-S8 is due to Speaker S7, but divergence in intonation is due to Speaker S8. This is somewhat in accordance with findings by Pardo and Louwerse et al. [18,24], in which an asymmetric accommodation occurs between speakers. In these studies, this asymmetry was found to be closely related to the role of the talker (information giver versus receiver). Our task, however, placed speakers in both giving and receiving roles. Other research similarly demonstrates that accommodation occurs to differing degrees in different measures and furthermore that convergence between interacting speakers can differ due to variation in social factors [20–22] as well as to individual variations in task involvement and attention [21,23], but, despite the explanatory ability of these sociolinguistic and attentional factors, they were not the main focus of our study. Rather, the fact that occasionally the identity of the more malleable speaker switched across measures suggests that factors like social dominance cannot be the sole cause of these asymmetries in degree of convergence. Likewise, the fact that Speaker S6 and Speaker S7 were the more malleable members of the dyads (Dyads S5-S6 & S7-S8), even though these dyads were sometimes diverging and sometimes converging, argues that the accommodation cannot be due to a less dominant member imitating a more dominant member [29,42]. This further implies that convergence in speech cannot be entirely attributed to an automatic or low-level ‘imitation-type’ function of perceived phonetic variants. A particularly intriguing possibility for the observed variability in accommodation across measures is that individuals may attend to different speech characteristics (either temporal or intonational, or both) in their interlocutor to a greater or lesser extent [48], thus affecting the measures in which accommodation is observed.

## Conclusions

In sum, this study utilizes a novel maze navigation task that allows for quasi-scripted speech from two interacting speakers so as to examine between-speaker accommodation. The experiment uses a dual magnetometer set-up to collect synchronized acoustic and articulatory kinematic data from the conversing speakers. The articulatory and acoustic data collected during this task is complemented by individual speaking conditions both before and after the dyad’s interaction, allowing for an examination of both the time course of speech convergence and persistence effects that may continue beyond the period of speaker interaction. Lastly, the study examines how prosodically salient positions, such as preceding a phrase boundary, may be differentially sensitive to accommodation forces when speakers are interacting in a cooperative task. The findings of convergence and divergence in prosodic measures have implications for understanding the realization of linguistic phrasal structure and its potential coupling across speakers. The findings on individual speaker malleability and the time course of accommodation have implications for understanding the cognitive forces underlying this mutual behavior in spoken language communication, in particular, providing novel evidence for accommodation at the level of cognitively specified motor control of individual articulatory gestures.

## References

[1] Schmidt RC, Carello C, Turvey MT. Phase transitions and critical fluctuations in the visual coordination of rhythmic movements between people. J Exp Psychol Hum Percept Perform. 1990;16: 227–247. 2142196 10.1037//0096-1523.16.2.227

[2] Clayton M, Sager R, Will U. In time with the music: the concept of entrainment and its significance for ethnomusicology. Eur Meet Ethnomusicol. 2005;11: 3–75.

[3] Vatikiotis-Bateson E, Barbosa AV, Best CT. Articulatory coordination of two vocal tracts. J Phon. Elsevier; 2014;44: 167–181.

[4] Cummins F. Gaze and blinking in dyadic conversation: A study in coordinated behaviour among individuals. Lang Cogn Process. 2012;27: 1525–1549.

[5] Levitan R, Gravano A, Willson L, Benus S, Hirschberg J, Nenkova A. Acoustic-Prosodic Entrainment and Social Behavior. The 2012 Conference of the North American Chapter of the Association for Computational Linguistics: Human Language Technologies. 2012. pp. 11–19.

[6] Pardo JS. Measuring phonetic convergence in speech production. Front Psychol. 2013;4: 1–5. 10.3389/fpsyg.2013.00001 23986738 PMC3753450

[7] Abney DH, Kello CT, Warlaumont AS. Production and Convergence of Multiscale Clustering in Speech. Ecol Psychol. 2015;27: 222–235.

[8] Abney DH, Paxton A, Dale R, Kello CT. Complexity matching in dyadic conversation. J Exp Psychol Gen. 2014;143: 2304–2315. 25285431 10.1037/xge0000021

[9] Tiede M, Bundgaard-Nielsen R, Kroos C, Gibert G, Attina V, Kasisopa B, et al Speech articulator movements recorded from facing talkers using two electromagnetic articulometer systems simultaneously. J Acoust Soc Am. 2010;11: 060007–060016.

[10] Levitan R, Hirschberg J. Measuring acoustic-prosodic entrainment with respect to multiple levels and dimensions. Proc Annu Conf Int Speech Commun Assoc INTERSPEECH. 2011; 3081–3084.

[11] Cohen Priva U, Edelist L, Gleason E. Converging to the baseline: Corpus evidence for convergence in speech rate to interlocutor’s baseline. J Acoust Soc Am. 2017;141: 2989–2996. 10.1121/1.4982199 28599520

[12] Nielsen K. Specificity and abstractness of VOT imitation. J Phon. Elsevier; 2011;39: 132–142.

[13] Babel M. Evidence for phonetic and social selectivity in spontaneous phonetic imitation. J Phon. Elsevier; 2012;40: 177–189.

[14] Brennan SE, Clark HH. Conceptual pacts and lexical choice in conversation. J Exp Psychol Learn Mem Cogn. 1996;22: 1482–1493. 8921603 10.1037//0278-7393.22.6.1482

[15] Branigan HP, Pickering MJ, Cleland AA. Syntactic co-ordination in dialogue. Cognition. 2000;75: 13–25. 10.1016/s0010-0277(99)00081-5 10771277

[16] Latif N, Barbosa A V., Vatiokiotis-Bateson E, Castelhano MS, Munhall KG. Movement coordination during conversation. PLoS One. 2014;9: 1–10. 10.1371/journal.pone.0105036 PMC4132081 25119189

[17] Tiede M, Mooshammer C. Evidence for an articulatory component of phonetic convergence from dual electromagnetic articulometer observation of interacting talkers. J Acoust Soc Am. 2013;19: 060138–060144.

[18] Pardo JS. On phonetic convergence during conversational interaction. J Acoust Soc Am. 2006;119: 2382–2393. 16642851 10.1121/1.2178720

[19] Pardo JS, Jay IC, Hoshino R, Hasbun SM, Sowemimo-Coker C, Krauss RM. Influence of Role-Switching on Phonetic Convergence in Conversation. Discourse Process. 2013;50: 276–300.

[20] Pardo JS, Urmanche A, Wilman S, Wiener J. Phonetic convergence across multiple measures and model talkers. Attention, Perception, Psychophys. 2017;79: 637–659. 10.3758/s13414-016-1226-0 27826946

[21] Sanker C. Comparison of Phonetic Convergence in Multiple Phonetic Features. Cornell Working Papers in Phonetics and Phonology. 2015; 60–75.

[22] Tamminga M, MacKenzie L, Embick D. The dynamics of variation in individuals. Linguist Var. 2016;16: 300–336. 10.1075/lv.16.2.06tam PMC6939640 31897370

[23] Abel J, Babel M. Cognitive Load Reduces Perceived Linguistic Convergence Between Dyads. Lang Speech. 2017;60: 479–502. 10.1177/0023830916665652 28915780

[24] Louwerse MM, Dale R, Bard EG, Jeuniaux P. Behavior matching in multimodal communication Is synchronized. Cogn Sci. 2012;36: 1404–1426. 10.1111/j.1551-6709.2012.01269.x 22984793

[25] Pardo JS, Urmanche A, Wilman S, Wiener J, Mason N, Francis K, et al A comparison of phonetic convergence in conversational interaction and speech shadowing. J Phon. Elsevier Ltd; 2018;69: 1–11.

[26] Bourhis R, Giles H. The language of intergroup distinctiveness In: Giles H, editor. Language, Ethnicity, and Intergroup Relations. London: Academic Press; 1977 pp. 119–135.

[27] Pardo JS, Gibbons R, Suppes A, Krauss RM. Phonetic convergence in college roommates. J Phon. 2012;40: 190–197.

[28] Geng C, Turk A, Scobbie JM, Macmartin C, Hoole P, Richmond K, et al Recording speech articulation in dialogue: Evaluating a synchronized double electromagnetic articulography setup. J Phon. Elsevier; 2013;41: 421–431.

[29] Nordham CA, Tognoli E, Fuchs A, Kelso JAS. How Interpersonal Coordination Affects Individual Behavior (and Vice Versa): Experimental analysis and adaptive HKB model of social memory. Ecol Psychol. Taylor & Francis; 2018;30: 224–249. 10.1080/10407413.2018.1438196 PMC7546216 33041602

[30] Gravano A, Benus S, Levitan R, Hirschberg J. Backward mimicry and forward influence in prosodic contour choice in Standard American. Proc Annu Conf Int Speech Commun Assoc INTERSPEECH. 2015; 1839–1843.

[31] Krivokapić J, Byrd D. Prosodic boundary strength: An articulatory and perceptual study. J Phon. 2012;40: 430–442. 10.1016/j.wocn.2012.02.011 23441103 PMC3579632

[32] Byrd D, Kaun A, Narayanan S, Saltzman E. Phrasal signatures in articulation. Pap Lab Phonol V Acquis Lex. 2000; 70–87.

[33] Byrd D. Relating prosody and dynamic events: comments on the papers by Cho, Navas, and Smiljanic. Pap Lab Phonol. 2006; 1–12.

[34] Byrd D, Choi S. At the juncture of prosody, phonology, and phonetics—The interaction of phrasal and syllable structure in shaping the timing of consonant gestures. Proceedings of Lab Phon X. 2006;10: 31–60.

[35] Turk A, Shattuck-Hufnagel S. Multiple targets of phrase-final lengthening in American English words. J Phon. 2007;35: 445–472.

[36] Fougeron C, Keating PA. Articulatory strengthening at edges of prosodic domains. J Acoust Soc Am. 1997;101: 3728–3740. 9193060 10.1121/1.418332

[37] Krivokapić J. Rhythm and convergence between speakers of American and Indian English. Lab Phonol. 2013;4: 39–65.

[38] Katsika A, Krivokapić J, Mooshammer C, Tiede M, Goldstein L. The coordination of boundary tones and its interaction with prominence. J Phon. 2014;44: 62–82. 10.1016/j.wocn.2014.03.003 25300341 PMC4185973

[39] Goldinger SD. Echoes of echoes? An episodic theory of lexical access. Psychol Rev. 1998;105: 251–279. 9577239 10.1037/0033-295x.105.2.251

[40] Nguyen N, Delvaux V. Role of imitation in the emergence of phonological systems. J Phon. Elsevier; 2015;53: 46–54.

[41] Pardo JS, Remez RE. The perception of speech 2nd ed Traxler MJ, Gernsbacher MA, editors. The Handbook of Psycholinguistics. San Diego: Elsevier; 2006.

[42] Remez RE. Analogy and Disanalogy in Production and Perception of Speech. Lang Cogn Neurosci. 2015;30: 273–286. 10.1080/23273798.2014.906636 25642428 PMC4310505

[43] Pardo JS. Expressing oneself in conversational interaction In: Morsella E, editor. Expressing Oneself/Expressing One’s Self: Communication, Cognition, and Identity. Hove, England: Psychology Press/Taylor & Francis; 2010 pp. 183–196.

[44] Boersma P, Weenink D. Praat: Doing phonetics by computer [Computer program]. Version 6.0.37 retrieved 3 February 2018 from http://www.praat.org/. 2018.

[45] Beckman ME, Elam GA. Guidelines for ToBI Labelling The Ohio State University Research Foundation 1993; 1–43.

[46] Byrd D, Saltzman E. Intragestural dynamics of multiple prosodic boundaries. J Phon. 1998;26: 173–199.

[47] Browman CP, Goldstein LM. Articulatory Phonology: An Overview. Phonetica. 1992;49: 155–180. 10.1159/000261913 1488456

[48] Yu ACL. Perceptual compensation is correlated with individuals’ “autistic” traits: implications for models of sound change. PLoS One. 2010;5: e11950 10.1371/journal.pone.0011950 20808859 PMC2924381

